# Video evidence of mountings by female-plumaged birds of paradise (Aves: Paradisaeidae) in the wild: Is there evidence of alternative mating tactics?

**DOI:** 10.1111/eth.13451

**Published:** 2024-02-19

**Authors:** Thomas MacGillavry, Claudia Janiczek, Leonida Fusani

**Affiliations:** 1 Konrad Lorenz Institute of Ethology, University of Veterinary Medicine, Vienna, Austria; 2 Department of Behavioural and Cognitive Biology, University of Vienna, Vienna, Austria

**Keywords:** alternative mating tactics, birds of paradise, homosexual behaviour, mating behaviour, sexual selection

## Abstract

The bewildering courtship phenotypes of male birds of paradise (*Paradisaedae*) represent a classic example of sexual selection through mate choice. While the majority of sexual selection studies have focused on either mate choice or intrasexual competition, males across a variety of taxa adopt alternative mating tactics as additional means of obtaining fertilization when they are otherwise unable to. For example, across various polygynous birds, subordinate males engage in sneak copulations, which may offset the fitness costs of prolonged subordinate periods. Despite exhibiting strong mating skews and male delayed plumage maturation, reports of sneak copulation in the birds of paradise are exceedingly rare. After reviewing an extensive video collection of courtship interactions, we found examples of mountings by female-plumaged birds in three birds of paradise species: the Western parotia *Parotia sefilata*, Carola’s parotia *Parotia carolae*, and the magnificent bird of paradise *Cicinnurus magnificus*. While homosexual mountings by females have been documented previously in Lawes’ parotia *P. lawesii*, adult males in the magnificent bird of paradise violently attacked intruding female-plumaged birds attempting to mount receivers, suggesting that they may be immature males engaging in alternative mating tactics. Overall, the rare video footage described here is suggestive of two fascinating, yet unexplored phenomena in polygynous birds: alternative mating tactics and female homosexual behaviour.

## 1 Introduction

The birds of paradise (family Paradisaeidae) comprise approximately 40 species endemic to New Guinea, the Northern Moluccas of Indonesia and subtropical Australia (Frith & Beehler, 1998). With the exception of a few species, males exhibit diverse and elaborate courtship phenotypes and represent a classic example of sexual selection through mate choice. The core paradisaeids (Irestedt et al., 2009) are polygynous and courtship typically takes place at display courts, which range from simple branches or dead tree stumps (e.g. riflebirds; genus *Ptiloris*), to elaborate arenas decorated with coloured seeds, insect frass, snake skins and mammal dung (e.g. parotias; genus *Parotia*; Frith & Beehler, 1998).

While such courts are usually frequented by courting adult males and females, they are also often visited by immature males which—unless partially moulted into their nuptial plumage—are typically indistinguishable from females in all polygynous species (LeCroy, 1981; LeCroy et al., 1980). However, since there is little opportunity for those males to successfully court females, the reason why immature males visit the courts of adults is unclear. For instance, immature males may improve the quality of their courtship displays by watching adults (i.e. sensorimotor learning; Spezie et al., 2022) and may enhance their immediate reproductive success by occasionally ‘stealing’ or ‘sneaking’ copulations from adults (Dougherty et al., 2022).

Such alternative mating tactics are widespread in polygynous mating systems, where intense female mate choice and intrasexual competition strongly skew mating success among males. Indeed, sneak copulation has been described in a wide variety of animal taxa, including arthropods (e.g. Wellington tree wētā [Nason & Kelly, 2020]), molluscs (e.g. cuttlefish *Sepia apama* [Hanlon et al., 2005]), actinopterygiid fishes (e.g. guppies *Poecilia reticulata* [Pilastro & Bisazza, 1999]) and squamates (e.g. side-blotched lizards *Uta stansburiana* [Sinervo & Lively, 1996]). In polygynous birds, sneak copulation has been described in several groups, including bowerbirds (Spezie & Fusani, 2022), manakins (Boyle & Shogren, 2019) and, most notably, the ruff *Calidris pugnax*—a lekking shorebird where ‘faeder’ males represent a genetically fixed sneak-mating morph (Jukema & Piersma, 2006; Küpper et al., 2016).

These, and a variety of other examples discussed elsewhere (e.g. see Dougherty et al., 2022) suggest that alternative mating tactics are widespread across the animal phylogeny and are of considerable importance in the evolution of mating systems. However, despite a long history of extensive field observations in the birds of paradise, mountings between female-plumaged individuals have only been reported twice in the wild (i.e. in Goldie’s Bird of Paradise *Paradisaea decora* [LeCroy et al., 1980] and Lawes’ parotia *Parotia lawesii* [Pruett-Jones & Pruett-Jones, 1990]).

The only other evidence of alternative mating tactics in this group comes from studies of captive lesser (*P. minor* [Laska et al., 1992]) and Raggiana’s bird of paradise (*P. raggiana* [Delacour & Mayer cited in Gilliard, 1969]), where females produced fertilized clutches when housed with immature males, though the conclusions derived from these captive studies have a limited applicability to wild systems. This suggests that immature males produce viable sperm before moulting into their definitive plumage—a phenomenon also previously documented in manakins (Foster, 1987). However, this remains to be demonstrated in wild populations.

While these past studies have provided at least tentative evidence of alternative mating tactics in the birds of paradise, video evidence of mountings by immature males in the wild has never been reported. Furthermore, in the 15 mountings between female-plumaged birds observed by Pruett-Jones and Pruett-Jones (1990) throughout a 3-year study of Lawes’ parotia, 13 cases involved individuals who were confirmed to be female. The two remaining cases involved unsexed birds that were suspected to be female. Overall, this represents an intriguing study system for female homosexual behaviour in birds, which is generally considered rare (MacFarlane et al., 2010).

In the present study, we expand upon these results with observations of mountings by female-plumaged birds in two additional parotia species—Carola’s parotia *P. carolae* and the Western parotia *P. sefilata*. While our conclusions are limited as the birds were not sexed, we further describe likely instances of sneak mounting in the magnificent bird-of-paradise *Cicinnurus magnificus*, a genus where mountings by female-plumaged birds have not yet been documented. Since displaying males in this species violently attacked intruding female-plumaged birds, we suspect that these individuals were immature males, though this warrants further study.

## 2 Methods

### 2.1 Acquisition of video footage

We reviewed 1758 videos of courtships in 30 species of birds of paradise between October 2021 and August 2022 covering all media available on the Cornell Lab of Ornithology Macaulay Library collected between 2003 and 2017 to search for instances of sneak copulation. This initial data set included footage showing only courtship behaviours. We then further filtered the video database to include only videos showing courtship displays (other courtship behaviours such as court-clearing were excluded) and where at least one female-plumaged individual was present in the frame, as this provides the minimum conditions for ‘sneak’ mountings to occur, and since other FP individuals may be present out of frame.

**Figure 1:**
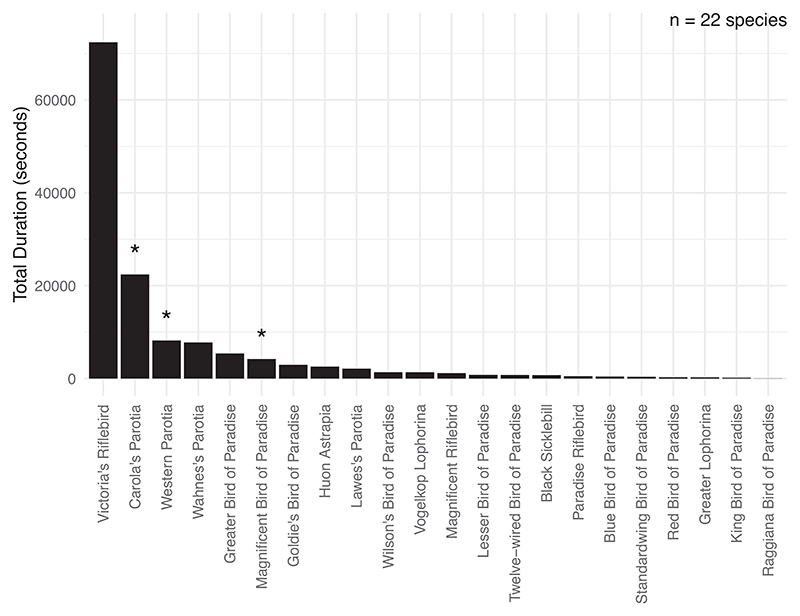
Total duration of video footage where at least one male was displaying and at least one receiver was attending the display across 22 species of birds of paradise. The remaining eight species for which video was available did not meet these criteria in the represented video footage. Asterisks demarcate species where mounting between female-plumaged birds was recorded. Mountings by female-plumaged birds were seen in species with better video coverage.

This yielded a final selection of 582 videos for 22 species encompassing approximately 1083 min of footage where (a) at least one courting male and (b) at least one female-plumaged individual was present at the court (see Figure 1). Furthermore, we only considered mountings as ‘successful’ if they involved the mounting bird pushing aside the tail of the recipient and making apparent cloacal contact. While the varying positions of birds in the video footage may have biased our delineation of ‘successful’ mountings, our data set included only one ambiguous case (at 01:46 in video number ML460178). However, since this attempted mounting occurred extremely rapidly (~6 frames from when the female-plumaged bird attempted mounting to when it was displaced by the courting adult male), it is unlikely to represent a successful mounting. All additional information for the archived footage can be found in the Macaulay Library (macaulaylibrary.org; Cornell University).

We further supplemented this data set with 845 videos encompassing approximately 1191 min of footage collected as part of our ongoing research project on Victoria’s riflebird *Ptiloris victoriae* in the Atherton tableland region in Far North Queensland, Australia (17.268° S, 145.627° E). We set up motion-activated camera traps at 18 display perches of adult males and video-recorded courtship display behaviour between August and November 2022 and 2023. The video footage was filtered as described above. See Figure 1 for a visual summary of the filtered video material for all species.

Riflebirds were captured with mist nets placed near display perches and banded with two to three plastic-coloured bands and a single-numbered metal band in order to discern between females and immature males. All 86 matings recorded were by adult males and unbanded female-plumaged individuals. Banding activities were conducted with the approval of the Australian Bird and Bat Banding Scheme (R-class banding authority number: 3662). Ethics approval was granted by the Animal Ethics Committee of the Department of Agriculture and Fisheries (AEC reference number: CA 2022/02/1589). Field activities at private properties and the Crater Lakes National Park (17.283°S, 145.625°E) were approved by the Queensland Wildlife and Parks Service (P-PTUKI-100257238; WA0045747).

## 3 Results

### 3.1 The occurrence of mountings by female-plumaged birds

Our search yielded 18 videos showing mounting by femaleplumaged individuals in *three* species: the magnificent bird of paradise *Cicinnurus magnificus*, Western parotia *Parotia sefilata* and Carola’s parotia *Parotia carolae*. Across these videos, a total of 29 mountings between female-plumaged individuals were observed and evidence of cloacal contact was seen in 13 out of 22 instances in Carola’s parotia (~60%), 2 out of 3 instances in the Western parotia (~67%), and 2 out of 4 instances (50%) in the magnificent bird-of-paradise (Table 1).

**Figure 2:**
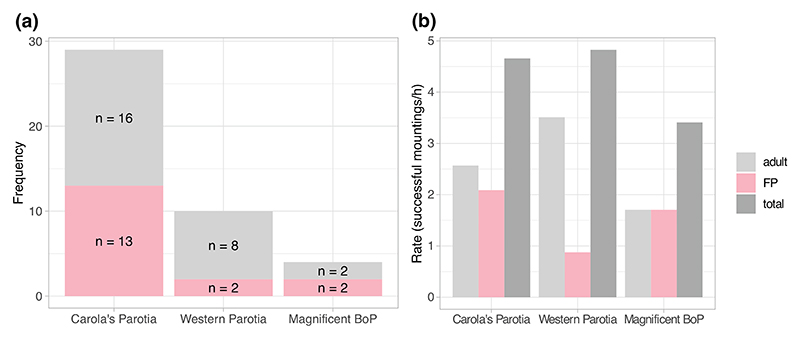
Plots showing the total frequencies of mountings observed (a) and the rates of mountings observed by adult males and female-plumage individuals per hour of footage (b). Only cases where cloacal contact appeared successful are included. ‘BoP’ = bird of paradise. Plots are purely for illustrative purposes as female-plumaged birds could not be individually identified and thus violate the assumption of independence of data required for formal statistical tests.

Based on the available video footage, the rate of successful mountings by female-plumaged individuals—measured as instances of mountings where there was evidence of cloacal contact per hour of courtship where at least one receiver is present—is 0.88 h^–1^ in the Western parotia (*n* = 2, 2.28 h of video), 2.09 h^–1^ in Carola’s parotia (*n* = 13, 6.23 h of video), and 1.71 h^–1^ in the magnificent bird of paradise (*n* = 2, 1.17 h of video). For comparison, rates of successful copulations by adult males in the same data set were observed to be 3.51 h^–1^ in the Western parotia (*n* = 8), 2.57 h^–1^ in Carola’s parotia (*n* = 16) and 1.71 h^–1^ (*n* = 2) in the magnificent bird of paradise (see Figure 2b). No sneak copulations were observed in our additional footage of Victoria’s riflebird.

### 3.2 General responses of adult males and recipients

**Figure 3:**
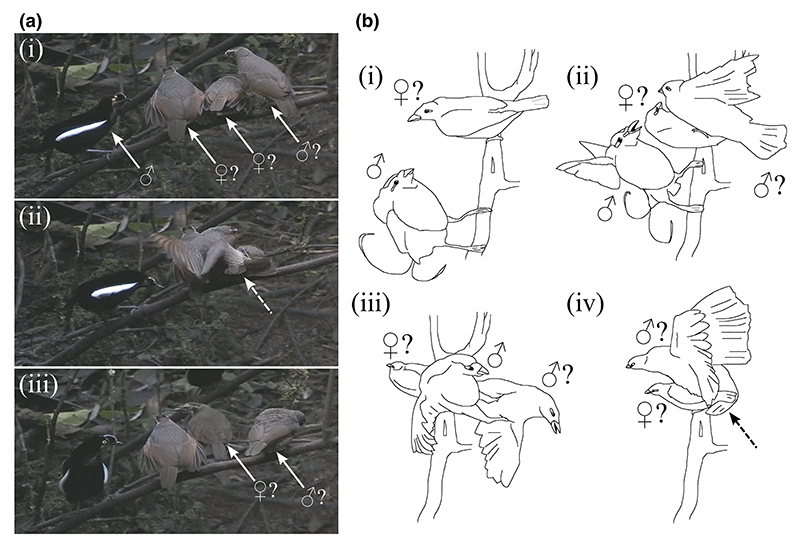
Examples of mountings by female-plumaged birds in Carola’s parotia (accession number: ML471843) and magnificent bird of paradise (accession number: ML460178; figure redrawn from video for clarity). Three female-plumaged birds are shown attending the court of an adult male at 16 s (a.i). The right-most female-plumaged bird is seen attempting to mount at 18 s (a.ii). Evidence of cloacal contact as described in the Methods section is indicated by a white arrow. The adult male does not appear to respond by 21 s and the female-plumaged bird returns to its former location on the receiver perch (a.iii). Panel B shows similar behaviour in the magnificent bird of paradise. First, an adult male displays to a receiver at 106 s (b.i). Immediately after, an female-plumaged individual attempts to mount the receiver (b.ii), to which the adult male responds by attacking at 107 s (b.iii). At 108 s, as the adult and female-plumaged bird fight, an additional female-plumaged bird is seen mounting with the receiver (apparently achieving cloacal contact) who remains perched on the display sapling (b.iv). Broken arrows indicate evidence of cloacal contact.

In the three species where mountings by female-plumaged birds were documented, the responses of courting males were markedly different (see Figure 3). In both *Parotia* species, the courting adult male did not appear to respond agonistically to female-plumaged mountings, while in both videos showing female-plumaged mounting in the magnificent bird of paradise, the courting male vigorously attacked the intruding FB birds. However, despite not responding agonistically, in two out of the three observed sneak mountings in the Western parotia, the dominant male immediately attempted to mount the female-plumaged bird that had previously been mounted.

In Carola’s parotia, in only three instances where an female-plumaged individual attempted to mount another did the recipient respond agonistically by either pecking at the mounting bird (accession number: ML471805) or by apparently ‘dumping’ the mounting bird off (accession numbers: ML471842 and ML471855). In each instance, the adult-plumaged male continued displaying, while in one video the male flies up to the audience perch following a sneak mounting (accession number: ML471861) but does not clearly respond to the mounting female-plumaged bird, which remains on the perch. More detailed descriptions of each video in which mountings by female-plumaged birds were seen as well as a catalogue of all filtered videos (see Table S1) are available in the supplementary information.

In both videos showing attempted sneak mountings in the magnificent bird of paradise, up to four female-plumaged birds were present around the courtship sapling. In both instances, an female-plumaged individual attempted to mount the receiver nearly coincidently with the dominant male, once it appeared receptive by wing-shivering. In one video (accession number: ML460178), two female-plumaged birds are seen sequentially mounting the receiver while the adult male vigorously attacks another female-plumaged bird.

## 4 Discussion and Conclusion

The combined observations gathered from the literature and reported in the present paper provide evidence of mountings by female-plumaged birds in five species of birds of paradise in the wild; Lawes’ parotia (Pruett-Jones & Pruett-Jones, 1990), Western parotia (this text), Carola’s parotia (this text), magnificent bird of paradise (this text) and Goldie’s bird of paradise (LeCroy et al., 1980). This latter case is the only known description of immature males (‘un-plumed’ males) mounting in the wild, though it is unclear whether evidence of cloacal contact was observed.

The most notable differences in the responses of adult males to sneak copulations were seen between the genera *Cicinnurus* and *Parotia*, where in the former, intruding males were violently attacked, whereas in the latter, the adult male either mounted immediately (e.g. *Parotia sefilata*) or did not appear to respond (e.g. *Parotia carolae*). While our sample sizes were small, the violent responses of courting adult male magnificent bird of paradise provide at least tentative evidence that the intruding female-plumaged birds were indeed immature males engaging in alternative mating tactics.

However, an intriguing alternative possibility is that these FB birds were females engaging in courtship disruption. For example in the Guianan cock-of-the-Rock *Rupicola rupicola*, 7.4% of the 3906 courtship visits by females observed were disrupted by other females (Trail & Koutnik, 1986). Given this hypothesis, it is unusual that disrupting females would attempt to mount females attending a displaying male rather than simply attacking them. Our observations are therefore much more consistent with the hypothesis that female-plumaged immature males engage in coercive alternative mating tactics in the magnificent bird of paradise. Indeed, in systems where sneak copulation by FP males has been confirmed, adult males tended to respond aggressively by fighting or chasing the intruding males away (Boyle & Shogren, 2019; Spezie & Fusani, 2022; Trail & Koutnik, 1986). This is indeed what was seen in the magnificent bird of paradise, where the courting male used its feet to lunge at the intruding female-plumaged birds in aggressive attacks (see Figure 2).

To convincingly suggest that immature males gain fitness benefits by subverting the mating decisions of females (by e.g., sneaking, stealing, or forcing copulations) researchers should genetically determine paternity among offspring. While accessing nests has proven challenging in birds of paradise (Frith & Beehler, 1998), better insight can be gained about the occurrence of alternative mating tactics in this taxon by monitoring banded populations and through the use of automated videos cameras placed at courts (Janisch et al., 2021). Intriguingly, rates of successful mountings by female-plumaged birds appear to be comparable to those of adult males in our data set (Figure 2), suggesting that the species discussed in the present paper are promising models to study homosexual behaviour and alternative mating tactics.

Sneak mating by female-plumaged individuals has numerous implications for the evolution of mating behaviour. For instance, sneak mating may generally be under negative frequency-dependent selection, as its reproductive benefits degrade when sneaker males are common because this selects for increasing the efficacy of resistance mechanisms in females and adult males (Engqvist & Taborsky, 2016; Giraldo-Deck et al., 2022; Rios-Cardenas et al., 2018). However, the costs of retaliation by adult males to attempted sneak mountings by competitors may further vary depending on the species’ social behaviour. In species where multiple females attend male courtships simultaneously, the benefits of attacking intruding males may be outweighed by the cost of other females leaving the display area. For instance, in the magnificent bird of paradise—where typically only a single receiver is perched on the display sapling at a time— adult males responded aggressively, while little retaliation was seen in the two parotia species, where up to six female-plumaged birds attended simultaneously (Carola’s parotia: $\bar{x}=2.65\pm 1.92$, *n* = 210 videos; Western parotia: $\bar{x}=2.03\pm 1.09$, *n* = 58 videos). There is, however, no clear benefit for adult males in responding agonistically to female homosexual behaviour, which may explain why no clear aggressive responses were observed in both Parotia species.

Despite more than a century of careful observation of wild birds of paradise (Frith & Beehler, 1998; Gilliard, 1969), mountings by female-plumaged individuals have rarely been documented. Our results corroborate the occurrence of this behaviour in the birds of paradise and show that it occurs across a variety of species with markedly different mating behaviours. While the birds of paradise represent a textbook example of sexual selection through mate choice and intrasexual competition, the observations presented here provide at least tentative evidence of sneak copulation in the wild. Alternatively, they may represent intriguing cases of female homosexual behaviour. Both hypotheses encompass poorly understood phenomena in the social systems of birds of paradise and warrant further research.

Very little remains known about alternative mating tactics in birds, including the reproductive benefits to immature males, costs to females and social dynamics between adult and immature males. Evidence of viable sperm production and successful reproduction by immature males in the wild would be extremely valuable to demonstrate the significance of sneak copulation in the birds of paradise. However, whether such fertilization could lead to the siring of offspring in the face of sperm competition with adults, remains unexplored.

While birds of paradise are seen as a system dominated by mate choice, strong skews in male reproductive success have likely driven the evolution of alternative mating tactics. While such behaviours have never been convincingly documented in the wild, the observations presented here should nonetheless encourage future field studies on alternative mating tactics and female homosexual behaviour in the birds of paradise.

## Supplementary Material

Data S1

Table S1

## Figures and Tables

**Table 1:** Accession numbers of videos showing mountings between female-plumaged birds and the associated notes and timestamps.

Macaulay Library accession number	Cloacal contact	Response of adult male	Response of recipient	Timestamp (s)	Date recorded
*Carola’s parotia*					
456734	Y	Continues displaying	None	48	01/10/2005
471794	Y	Continues displaying	None	136	21/11/2000
471805	Y	Continues displaying	None	24	21/11/2000
	Y	Continues displaying	None	43	
	N	Continues displaying	Pecks at male	47	
471806	Y	Continues displaying	None	4	21/11/2000
471808	Y	Continues displaying	None	0	24/11/2000
471842	Y	Continues displaying	Dumps mounting bird	221	24/11/2000
471843	Y	Continues displaying	Hops to adjacent perch	17	24/11/2000
471851	Y	Continues displaying	None	153	24/11/2000
471854	Y	Continues displaying	None	56	24/11/2000
	N	Continues displaying	None	56	
471855	Y	Continues displaying	Dumps mounting bird	117	24/11/2000
	Y	Continues displaying	None	137	
	N	Continues displaying	None	143	
	Y	Continues displaying	None	162	
471857	N	Continues displaying	None	54	24/11/2000
	N	Continues displaying	None	55	
471858	N	Continues displaying	None	33	24/11/2000
	N	Continues displaying	None	40	
471861	N	Flies up to perch	None	120	24/11/2000
	N	None	None	146	
*Western parotia*					
468875	N	Continues displaying	Flies away	172	28/11/2004
468916	Y	Immediately copulates with females	None	148	04/12/2004
468926	Y	Immediately attempts to mount female	Falls from perch	82	04/12/2004
*magnificent bird of paradise*					
460173	N	Attacks	None	47	30/09/2010
460178	N	Attacks	None	106	30/09/2010
	Y	Continues attacking	None	108	
	Y	Continues attacking	None	110	

## Data Availability

All video footage described in the present article is available on the Macaulay Library at the Cornell Lab of Ornithology (https://www.macaulaylibrary.org/). Accession numbers are given in the main text.
