# Supplementary material for: Video evidence of mountings by female-plumaged birds of paradise (Aves: Paradisaeidae) in the wild: Is there evidence of alternative mating tactics?
Source: Ethology. Author manuscript; Available in PMC 2024 Dec 9. (PMC7617153; doi:10.1111/eth.13451)
Supplement: Data S1 [file EMS197396-supplement-Data_S1.docx]

**Online supplementary information for:**

**Video Evidence of Mountings by Female-Plumaged Bird-of-Paradise (*Aves*: *Paradisaeidae*) in the Wild: Is There Evidence of Alternative Mating Tactics?**

Thomas MacGillavry^1^, Claudia Janiczek^2^, Leonida Fusani^1,2^

*^1^Konrad Lorenz Institute of Ethology, University of Veterinary Medicine, Vienna, Austria*

*^2^Department of Behavioural and Cognitive Biology, University of Vienna, Vienna, Austria*

**Detailed descriptions of sneak copulation—Carola’s parotia *Parotia carolae*.**

**ML456734**: A FB is seen mounting another at 00:48 and appears to achieve cloacal contact at 00:50. The adult male does not appear to respond and performs the *swaying bounce* display throughout (Scholes, 2006).

**ML471794**: A FB is seen mounting another at 02:16 and appears to achieve cloacal contact at 02:18. The adult male does not appear to respond and performs the *head tilting* display throughout (Scholes 2006). Three additional adult males are present at the court.

**ML471805**: A FP bird is seen mounting another at 00:23 and appears to achieve cloacal contact at 00:24. The adult male does not appear to respond and performs the *head tilting* display throughout (Scholes 2006).

**ML471806**: A FP bird is seen mounting another at 00:03 and appears to achieve cloacal contact at 00:05. This individual attempts to mount the same FP bird at 00:23, but fails as the recipient moves away.

**ML471808**: A FP bird mounts another at 00:00 and appears to achieve cloacal contact at 00:01. The adult male hops onto the same perch at 00:03, but does not appear to behave agonistically. At the same time, another FP bird mounts another, but does not appear to achieve cloacal contact before dismounting at 00:10.

**ML471842:** A FP bird mounts another at 03:38 and again at 03:40 following apparent cloacal contact at 03:42. The mounted birds appears to dump the mounting bird off at 03:43. The adult male does not appear to respond and performs the *head tilting* display throughout (Scholes 2006).

**ML471843**: A FP bird is seen mounting another at 00:16 and appears to achieve cloacal contact at 00:18. The adult male does not appear to respond and performs the *head tilting* display throughout (Scholes, 2006).

**ML471851**: A FP bird is seen mounting another at 02:32 and appears to achieve cloacal contact at 02:34. Immediately after, the mounted bird hops down from the perch. The adult male does not appear to respond and performs the *head tilting* display throughout (Scholes, 2006).

**ML471854**: A FP bird is seen mounting another at 00:56 and appears to achieve cloacal contact at 00:57. The adult male does not appear to respond and performs the *swaying bounce* display throughout (Scholes 2006). Another adult male interrupts the display at 01:03.

**ML471855**: A FP bird is seen mounting another at 01:57 and appears to achieve cloacal contact at 01:58. The recipient appears to immediately throw the mounting bird off, who attempts to mount another FP bird at 02:17, apparently achieving cloacal contact at 02:18. Several seconds later at 02:21, a second FP bird mounts another, but does not achieve cloacal contact and dismounts at 02:25. An attempted mounting is seen at 02:39, but fails as the recipient hops to another branch. Yet another mounting is seen at 02:42, with apparent cloacal contact at 02:43. The adult male performs the *swaying bounce* display throughout and does not appear to respond (Scholes, 2006) and chases another bird perched mostly out of frame at 03:24, but this appears to be another adult male. Overall, five sneak mountings—of which three involved cloacal contact—by two FP birds were seen throughout the 3 minute and 32 second duration of this video.

**ML471857**: At 00:53 a FP bird mounts another perched on a vertical branch but fails to achieve cloacal contact. At 00:54 a second FP bird mounts another but also fails to achieve cloacal contact. While both sneak mountings occur, the courting male performs the *head tilting* display and does not appear to respond (Scholes, 2006).

**ML471858**: At 00:40 a FP bird mounts another perched on a vertical branch but fails to achieve cloacal contact. The courting male performs the *swaying bounce* display and does not appear to respond (Scholes, 2006). At 05:32, the displaying adult male successfully copulates.

**ML471861**: At 01:59 a FP bird mounts another perched on a vertical branch while the displaying male performs the *hop-and-shake* display (Scholes, 2006), but fails to achieve cloacal contact. The same individual mounts another FB bird at 02:26 now with the courting adult male perched directly beside, but again does not make cloacal contact.

**Detailed descriptions of sneak copulation—Western parotia *Parotia sefilata.***

**ML468875**: At 02:53 a FP bird mounts another while courting adult male performs the *ballerina dance* display (Scholes 2008). The mounted bird then drops from the perch at 02:54 apparently startling the adult male, which then performs the *hops-across-court* display (Scholes 2008).

**ML468916**: A FP bird mounts another at 02:04 followed immediately by the courting male flying up to the perch, pushing the mounting FP bird aside, and mounting the presumed female. It is unclear whether this mounting involved cloacal contact since the modified flank plumes obscured a clear view of the tail positions of both birds. The mounting FP bird remains perched directly next to the copulating pair throughout and is not chased away by the adult male.

**ML468926**: A FP bird is seen mounting another at 01:21 and appears to achieve cloacal contact at 01:24. The adult male responds by immediately flying up to the perch and appears to attempt to mount the mounted FP bird, but fails as the recipient leaves the perch. No agonistic behaviour towards the mounting FP bird was observed.

**Detailed descriptions of sneak copulation—Magnificent bird-of-paradise *Cicinnurus magnificus.***

**ML460173**: At 00:47, the courting adult male approaches the receiver at which point two FP birds appear to attempt copulation, joined by a third at 00:48. One FP bird successfully mounts the receiver, but it is unclear whether cloacal contact is made. Overall, it appears that four FP birds were present around the court in addition to the visiting receiver. These individuals all appeared to attempt to copulate with the visiting female at the same time as the courting adult male, which responded by attacking them.

**ML460178**: A FP bird is seen attempting to mount another on the courtship sapling at 01:46 but is aggressively removed by the adult male. While the adult male is engaged in combat, another FP bird mounts and appears to achieve cloacal contact with the receiver at 01:48, after which another FP bird attempts to mount, causing both to fall from the sapling while yet another FP bird mounts and appears to achieve cloacal contact with the receiver at 01:50. The adult male is then seen chasing away the remaining FP birds and exits the court at 01:55.

**Table S1.** All videos showing at least one adult male displaying at at least one female-plumaged individual and associated metadata. All videos, other than those of Victoria's Riflebor collected by T. MacGillavry, are available in the Macaulay Library (Cornell Lab of Ornithology).
